# Photoelectric tunable-step terahertz detectors: a study on optimal antenna parameters, speed, and temperature performance

**DOI:** 10.1515/nanoph-2023-0864

**Published:** 2024-02-01

**Authors:** Ran Chen, Ruqiao Xia, Jonathan Griffiths, Harvey E. Beere, David A. Ritchie, Wladislaw Michailow

**Affiliations:** Cavendish Laboratory, University of Cambridge, CB3 0HE Cambridge, UK; Swansea University, Singleton Park, Sketty, Swansea SA2 8PP, UK

**Keywords:** two-dimensional electron gas, in-plane photoelectric effect, photoelectric tunable-step detector, far-infrared detection

## Abstract

Field effect transistors have shown promising performance as terahertz (THz) detectors over the past few decades. Recently, a quantum phenomenon, the in-plane photoelectric effect, was discovered as a novel detection mechanism in gated two-dimensional electron gases (2DEGs), and devices based on this effect, photoelectric tunable-step (PETS) THz detectors, have been proposed as sensitive THz detectors. Here, we demonstrate a PETS THz detector based on GaAs/AlGaAs heterojunction using a dipole antenna. We investigate the dependence of the in-plane photoelectric effect on parameters including the dimensions and the operating temperature of the device. Two figures of merit within the 2DEG, the maximum electric field and the radiation-induced ac-potential difference, are simulated to determine the optimal design of the PETS detector antenna. We identify the optimal antenna gap size, metal thickness, and 2DEG depth, and demonstrate the first PETS detector with a symmetric dipole antenna, which shows high-speed detection of 1.9 THz radiation with a strong photoresponse. Our findings deepen the understanding of the in-plane photoelectric effect and provide a universal guidance for the design of future PETS THz detectors.

## 1 Introduction

Terahertz (THz) waves refer to electromagnetic waves within the frequency range *f* = 0.1–10 THz. Lying in the frequency gap between conventional electronic and photonic regions, THz waves have been one of the less explored research areas for a long time [1], [2], [3], due to a lack of efficient, cheap, and easy-to-use sources and detectors, and the term “THz gap” has been coined to describe this technological void. Today, the interest in the THz region is greatly fueled by its unique applications in various areas, including medical imaging [4], [5], ultrafast wireless communication [6], [7], non-destructive testing [8], [9], pollutant gas detection [10], defense and security scanning [11], [12].

To shrink the “THz gap” and bring all of the applications to fruition, many efforts have been devoted over the last few decades to finding effective THz detectors [13], [14]. One of the promising detection systems for THz radiation is field effect transistors (FETs) based on two-dimensional electron gases (2DEGs). The idea of using field effect transistors as THz detectors gained interest since the proposition of plasmonic mixing [15] in 2D electron systems as a mechanism to generate a THz photoresponse. Since then, field effect transistors on the basis of gallium arsenide [16], [17], [18], silicon [19], [20], [21], and gallium nitride [22], [23] have been explored as THz detector devices utilizing resistive mixing, distributed resistive mixing, and plasmonic mixing as the underlying mechanisms [24], [25]. With the advent of 2D materials, a number of THz detectors exploiting the photo-thermoelectric effect [26], [27] have been realized, including devices with a single antenna [28], [29], but also in an asymmetric dual grating gate geometry [30], [31]. FET-based detectors have shown large responsivities in the sub-THz range, such as 4.9 kV/W in an InGaAs FET at 0.8 THz [18] or 2.2 kV/W in an InAlAs/InGaAs/InP FET at 1 THz [32]. However, it proves more challenging to realize high sensitivity FET-based THz detectors in the region above 1–2 THz. For example, in silicon-based FETs, responsivities of 30 V/W at 2.9 THz [25], 230 V/W at 3.1 THz [33], 75 V/W at 4.75 THz [20] have been observed, and 50 V/W at 3.4 THz in graphene-based FETs [29]. Other mechanisms responsible for the generation of a THz photoresponse have also been demonstrated, such as collision-less electron heating [34], photon-assisted tunneling [35], bolometric [36], and photovoltaic effects [37].

In 2022, a novel, quantum, collision-free mechanism, the in-plane photoelectric effect (IPPE), was discovered [38], [39]. It was shown to produce a giant photoresponse to THz radiation in a bowtie-shaped 2DEG-based photoelectric tunable-step (PETS) THz detector. As was demonstrated in Ref. [38], the in-plane photoelectric effect can produce a much stronger photoresponse to THz radiation compared with other existing mechanisms in 2D electron systems, such as plasma-wave mixing, making it a potential new approach for THz detection.

**Figure 1:**
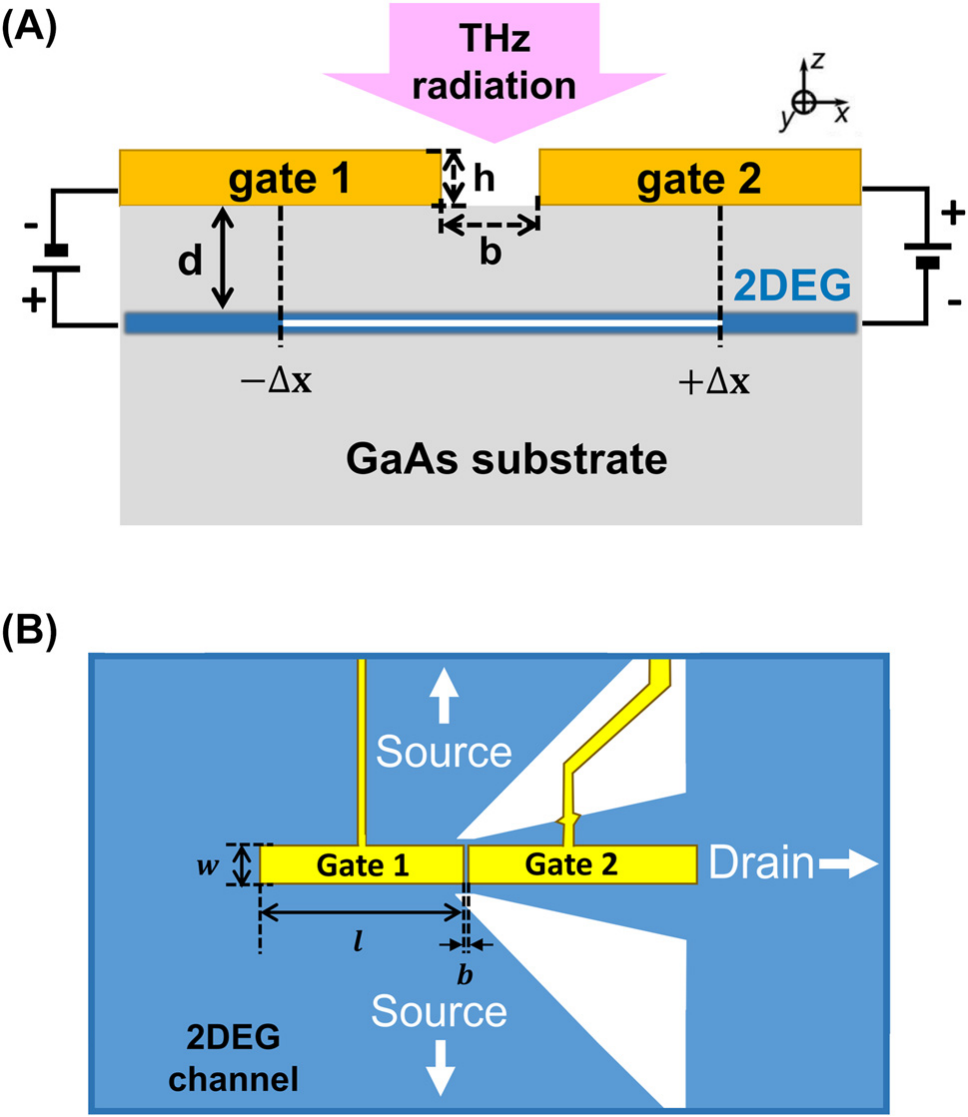
Detector design. (A) Side view of a dual-gated PETS THz detector based on a GaAs/AlGaAs heterojunction in the *y* = 0 plane. The parameters *d*, *b* and *h* represent the 2DEG depth, the gap size between the two gates, and the gate thickness, respectively. The white line within the 2DEG layer is the integration range for the radiation-induced ac potential difference. (B) Schematic diagram of the dipole antenna at the top of the detector. The architecture of the device is a 2DEG-based dual-gate field effect transistor with source and drain contacts, and two independent gates. The blue area is a mesa which confines the 2DEG underneath, while in the white area the 2DEG is etched away. The parameters *l* and *w* are the gate length and gate width of a dipole antenna arm.

In this work, a dual-gated 2DEG-based PETS THz detector with a dipole antenna on top of a GaAs/AlGaAs heterojunction, as shown in Figure 1, is utilized to carry out a systematic study on the in-plane photoelectric effect. By applying different voltages to the two independent gates, the bottom of the conduction band shifts under the left and right gates, respectively, and an electrically tunable potential step is artificially created within the 2DEG channel. Without incident illumination, there is no net current flow. However, when the dual-gated device is exposed to incident THz radiation, the dipole antenna on top will couple the incident THz radiation to the 2DEG by amplifying the electric field within the gap between two gates. Electrons absorb photons in the vicinity of the artificially created potential step, which leads to a net electron flow within the 2DEG from the higher-density region to the lower-density region, in other words, onto the potential step. This results in a dc photocurrent response under zero source-drain bias via the in-plane photoelectric effect.

To optimize PETS THz detectors for high responsivity, we investigate the dependence of the THz photoresponse on various parameters in this work using both numerical simulations and experimental measurements. Our dipole antenna PETS detector design represents a model system which allows the optimal antenna dimensions to be derived in a way that can be universally applied to any type of future PETS THz detectors. We also measure the speed of the detector as well as the photoresponse dependence as a function of operating temperature, and demonstrate successful PETS detection at significantly higher temperatures than in Ref. [38].

## 2 Simulation

### 2.1 Simulation method

**Figure 2:**
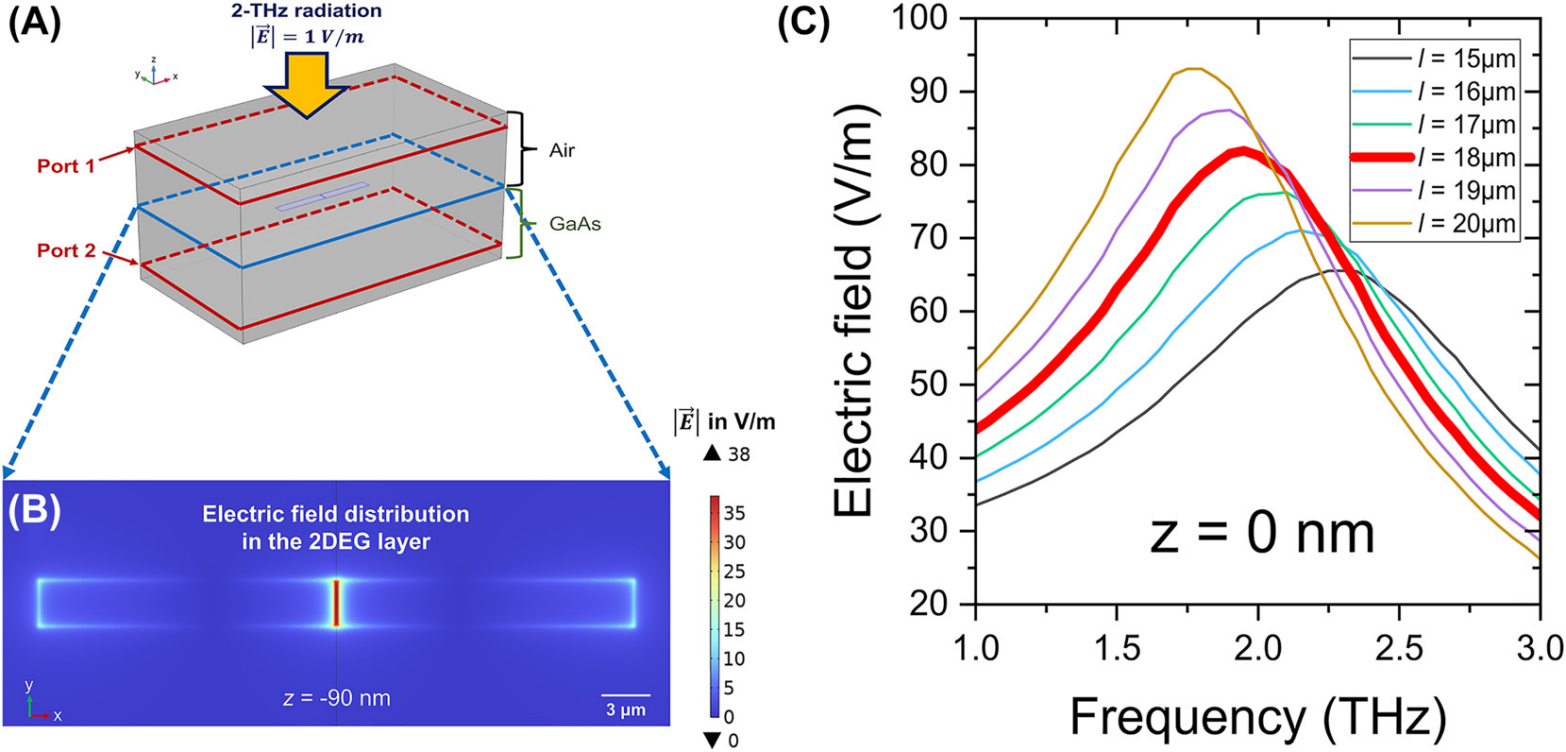
Simulation configuration. (A) Schematic diagram of the 3D simulation model. The area above the work plane with a dipole antenna is air with a relative permittivity of 1. The substrate below the work plane is GaAs with a 12.6 relative permittivity at liquid helium temperature [40]. A 2-THz plane wave, normalized to 1 V/m, is incident onto the system from port 1 in the air region. The layers above port 1 and below port 2 are perfectly matched layers which can eliminate reflections from the surfaces. (B) Simulated electric field distribution in the 2DEG layer with 2DEG depth *d* = 90 nm, gap size *b* = 190 nm, dipole length *l* = 18 μm, dipole width *w* = 3 μm and gate thickness *h* = 100 nm. (C) Simulated spectra of the average electric field amplitude within the gap between two gates. The dipole length *l* is swept from 15 to 20 μm while the dipole width *w* is fixed to 3 μm, the gap size *b* is set to 190 nm and the gate thickness *h* is 100 nm.

To determine the optimal dimensions of the PETS THz detector, the resonant frequency and the electric field distribution within the 2DEG channel are simulated with the commercial finite-element simulation software COMSOL Multiphysics. As shown in Figure 2(A), the device is fabricated on a GaAs substrate with a relative permittivity of 12.6 at liquid helium temperature [40]. The gold dipole antenna extruding from the top surface is designed to confine the incident THz radiation and amplify the electric field within the 2DEG. To describe the frequency-dependent conductivity of gold, $\sigma \left(f\right)$ , Drude model is utilized,

(1) $$\sigma \left(f\right)=\frac{\varepsilon_{0}{\left(2\pi f_{p}\right)}^{2}\tau }{1+i2\pi f\tau },$$

where *ɛ*_0_ is the vacuum permittivity, *f*_*p*_ is the plasma frequency of gold, and *τ* is the scattering time. We use *f*_*p*_ = 2.043 × 10^15^ Hz and *τ* = 1.4 × 10^−14^ s [41]. A 2-THz plane wave with a normalized 1 V/m electric field is incident onto the system. The gate length *l* and the gate width *w* are set to 18 μm and 3 μm, respectively, to tune the resonant frequency of the device to 2 THz, as illustrated in Figure 2(C). The THz bandwidth of the antenna is about 1 THz (1.4 THz – 2.45 THz), read out as the full width at half maximum of ${\left|E\right|}^{2}\left(f\right)$ . Only the central *y* = 0 plane shown in Figure 1(A) is studied in the simulation due to the symmetry of the architecture.

Two figures of merit determining the THz photoresponse, *E*_x,max_ and *U*_ac,x_, are analyzed to determine the optimal antenna design of a PETS THz detector:

(a) Maximum electric field within the 2DEG
(2) $$E_{\text{x},\max}\left(b,d,h\right)={\text{max}}_{x}\left(E_{\text{x}}\left(x,z=-d;b,h\right)\right)$$
(b) Radiation-induced ac-potential difference within the 2DEG
(3) $$U_{\text{ac},\text{x}}\left(b,d,h\right)=\overset{\Delta x}{\underset{-\Delta x}{\int }}E_{\text{x}}\left(x,z=-d;b,h\right)\text{d}x$$
  Here, the integration length, as illustrated by the white line in Figure 1(A), 2Δ*x* = 18 μm (i.e. from −9 μm to 9 μm) is set equal to the gate length *l*.

In the theoretical limit of an infinitely sharp potential step, i.e. *b* → 0, the IPPE photocurrent can be calculated by analytically solving the time-dependent Schrödinger equation in the first-order perturbation theory, treating the THz-induced time-dependent potential as the perturbation. The expression of *I*_ph_ is proportional to $U_{\text{ac},\text{x}}^{2}$ [38], [39]:

(4) $$I_{\text{ph}}=ef{\left(\frac{eU_{\text{ac},\text{x}}}{\hbar \omega }\right)}^{2}\left[2\sqrt{\frac{\hbar \omega }{E_{w}}}J\left(\frac{\mu_{\text{L}}}{\hbar \omega },\frac{\mu_{\text{R}}}{\hbar \omega }\right)\right]$$

Here, *J* is a universal dimensionless function depending on dimensionless parameters, $\frac{\mu_{L}}{\hbar \omega }$ and $\frac{\mu_{\text{R}}}{\hbar \omega }$ . *μ*_L_ and *μ*_R_ are the local chemical potentials under the left and right gates. In the theory, a simplified approximation was used, where *U*_ac,x_ was assumed to be product of the average electric field in the 2DEG layer within the gap region, multiplied with the gap size *b*. However, in a real device, $E_{\text{x}}\left(x,z=-d\right)$ has a more complicated form, which we obtain here from simulations. Hence both the maximum electric field *E*_x, max_, Eq. (2), as well as the integrated ac potential $U_{\text{ac},\text{x}}$ , Eq. (3), need to be considered. *E*_x, max_ as the derivate of the ac potential also represents the sharpness of the THz-induced oscillating ac potential step within the gate gap, and the sharper it is, the more suitable is the theoretical model of the potential step as infinitely sharp, which was used to derive Eq. (4).

Here, the dependencies of *E*_x,max_ and *U*_ac,x_ on three parameters of the architecture, 2DEG depth *d*, gap size *b*, and gate thickness *h*, are simulated to find out the optimal dimensions of the PETS THz detector.

### 2.2 Influence of the gap size *b*

**Figure 3:**
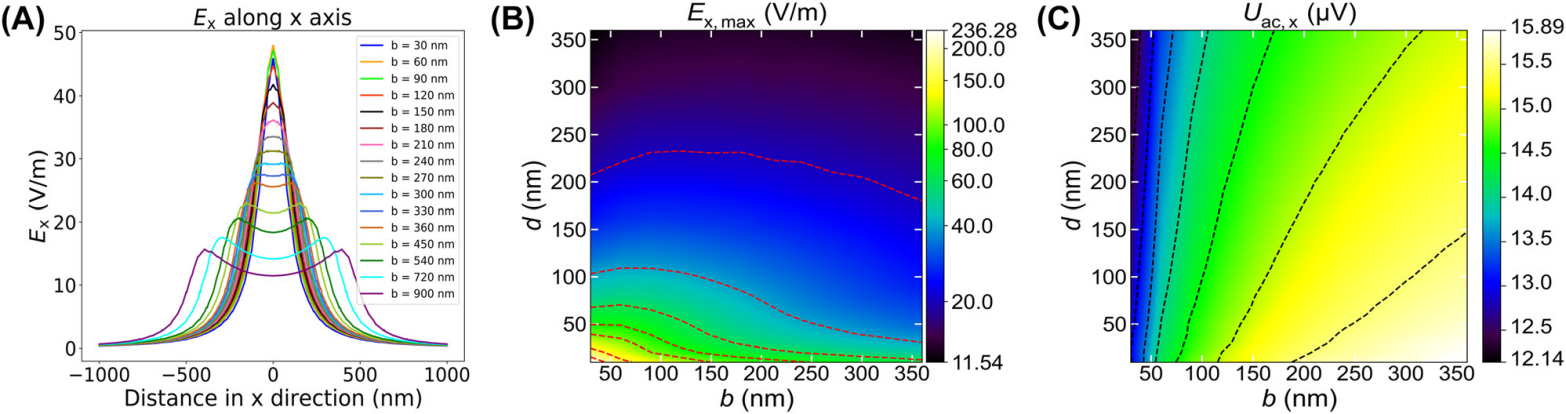
Simulation results of a dipole-antenna PETS THz detector with dipole length *l* = 18 μm, dipole width *w* = 3 μm and gate thickness *h* = 100 nm. (A) Distribution of *E*_x_ along the *x*-direction in the 2DEG layer with a 2DEG depth *d* = 90 nm. (B) Maximum *E*_x_, *E*_x,max_, within the 2DEG channel as a function of the gap size *b* and the 2DEG depth *d*, note the logarithmic color scale. (C) Radiation-induced ac-potential difference, *U*_ac,x_ (*b*,*d*), calculated by integrating *E*_x_ along the *x* direction with Δ*x* = 9 μm as a function of the gap size *b* and the 2DEG depth *d*.

The distribution of the x-component of the electric field along the *x*-direction at a 2DEG depth *d* = 90 nm, $E_{\text{x}}\left(d=90\;\text{nm}\right)$ , is shown in Figure 3(A). As can be seen, the incident electric field is focused and greatly amplified within the gap of the two gates by the dipole antenna. To get a sharp, single-peak *E*_x_-field, the gap size *b* should be smaller than a certain value *b*_max_, $b<b_{\max}\left(d\right)$ , beyond which the peak splits in two. Here, with a 2DEG located 90 nm below the top surface, $b_{\max}\left(d=90\;\text{nm}\right)\approx 300\;\text{nm}$ .

**Figure 4:**
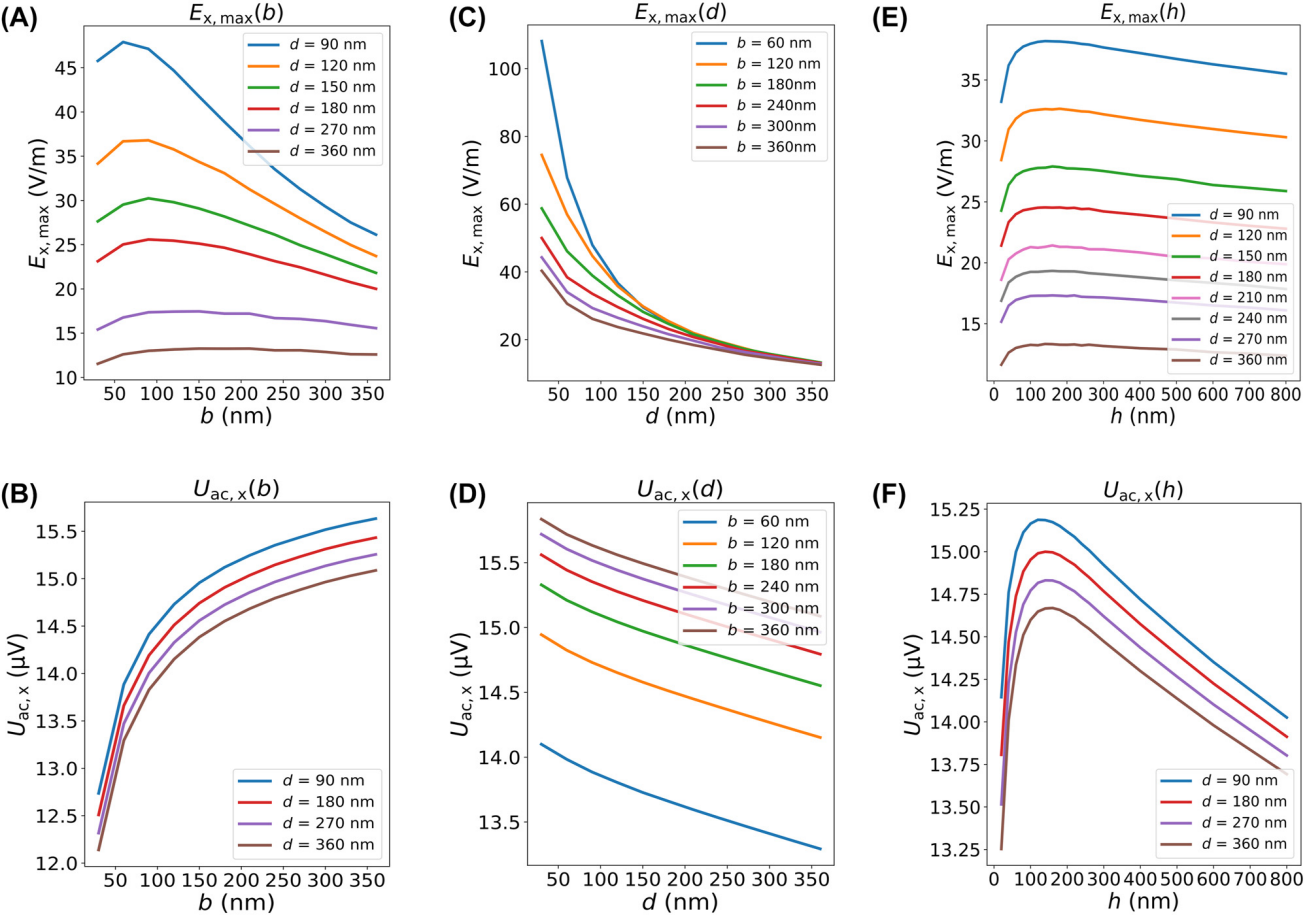
Detailed simulation of the dependence of *E*_x,max_ and *U*_ac,x_ on the gap size *b* (A, B), the 2DEG depth *d* (C, D), and the gate thickness *h* (E, F). The gate thickness *h* for figures (A–D) is 100 nm. The gap size *b* for figures (E–F) is 190 nm. The length and width of the metal pads of the dipole antenna are fixed to 18 μm and 3 μm, respectively.

Figure 3(B) and (C) are the simulated 2D maps of *E*_x, max_ and *U*_ac,x_ as functions of gap size *b* and 2DEG depth *d*. To make both *E*_x, max_ and *U*_ac,x_ as high as possible for a strong photoresponse, we consider the lower left corners, where both *b* and *d* are small. The detailed investigation of $E_{\text{x},\max}\left(b\right)$ in Figure 4(A) shows that *E*_x, max_ increases first and then drops down with an increasing *b*. The optimal *E*_x, max_ is reached when the gap size *b* is on the same order of magnitude and slightly smaller than *d*, which is consistent with the result in Figure 3(A) where the highest $E_{\text{x}}\left(d=90\;\text{nm}\right)$ is achieved at *b* = 60 nm, approximately around *b* ≈ 2*d*/3. The detailed dependence of *U*_ac,x_ on the gap size *b* is illustrated in Figure 4(B). *U*_ac,x_ grows slowly as *b* increases from 30 nm to 360 nm, and changes by less than 3 % of the maximum beyond *b* = 200 nm. Therefore, the effect of *b* on *E*_x, max_ is much greater than on *U*_ac,x_.

The analysis of the *E*_x, max_- and *U*_ac,x_-dependence on *b* and *d* allows us to conclude:

(a) The gap size should be smaller than a certain value *b*_max_, $b<b_{\max}\left(d\right)$ , to obtain a sharp, single-peak *E*_x_-field. For a 2DEG layer 90 nm below the top surface, $b_{\max}\left(d=90\;\text{nm}\right)\approx 300\;\text{nm}$ .
(b) Both *E*_x, max_ and *U*_ac,x_ are around the optimal values when the gap size *b* is on the same order of magnitude and slightly smaller than *d*, approximately *b* ≈ 2*d*/3.

### 2.3 Influence of the 2DEG depth *d*

As shown in Figure 4(C) and (D), both *E*_x, max_ and *U*_ac,x_ decrease with an increasing 2DEG depth *d*. Thus, the 2DEG channel should be located as close to the top surface as possible. In practice, below a certain thickness of the dielectric barrier, the gate leakage will increase. In order to exclude gate leakage as a possible parameter affecting the photoresponse, we use wafers with a safely large 2DEG depth of ca. *d* = 90 nm, which at the same time supports a high mobility of the 2DEG in the channel.

### 2.4 Effect of the gate thickness *h*

The gold dipole antenna at the top surface of the device is described using the Drude model according to Eq. (1). The dependencies of *E*_x, max_ and *U*_ac,x_ on the gate thickness *h* are simulated in Figure 4(E) and (F), respectively. As *h* increases, both *E*_x, max_ and *U*_ac,x_ rise up first and then drop down, with their largest values achieved around *h* = 150 nm, with a weak dependence on *h*. The lines with different 2DEG depth in Figure 4(E) and (F) reveal that the optimal value of *h* is independent of *d*. Therefore, regardless of where the 2DEG channel is located, both figures of merit reach a flat maximum with a gate thickness of around 150 nm.

### 2.5 Fabry–Perot interference

As shown in Figure 2(A), we place the second port inside of the GaAs material, which enables us to study the antenna characteristics without influence of the Fabry-Perot effect within the substrate material. However, antennas on real samples processed on a finite substrate may be exposed to a different electric field due to the wave interference within the substrate material with refractive index *n*. To estimate this effect, we analytically calculate the ratio of the electric fields present at the top surface of a substrate with thickness *D*, and at the surface of an infinite substrate. This ratio is given by:

(5) $$f\left(n,k,D\right)=1-\frac{2n\left(n-1\right)}{{\left(n-1\right)}^{2}-{\left(n+1\right)}^{2}e^{\mathrm{2ikDn}}}$$

With the refractive index *n* = 3.55 for GaAs [40], the maxima and minima of this function have values of 2.275 and 0.334, respectively. If we assume that the Fabry–Perot effect modifies the electric field present at the surface, which is then further amplified by the antenna, we can estimate that the photoresponse can be changed by up to the square of these values, i.e. multiplied by a value between 5.18 and 0.112. This yields opportunities for a further enhancement of the photoresponse of PETS detectors, which can be realized by a tailored choice of the substrate thickness or operation wavelength of the THz source. It also represents an additional factor that needs to be considered when comparing the experimentally measured photoresponse with the theoretically expected value.

## 3 Experiment

### 3.1 Samples and setup

Based on the simulation results stated above, we fabricate a dipole antenna PETS THz detector with 2DEG depth *d* = 90 nm, gap size *b* = 190 nm, and gate thickness *h* = 175 ± 5 nm. We measured the gap size using scanning electron microscopy, and confirmed the gate thickness by atomic force microscopy. While a smaller gap size would yield a larger maximum electric field based on the simulation, we use *b* = 190 nm, as the achievable gap size is limited by our current fabrication process. The resonant frequency is tuned to 2 THz by fixing the gate length and width to 18 μm and 3 μm, respectively. The simulated electric field distribution of this device in the 2DEG layer, *d* = 90 nm under the surface, is shown in Figure 2(B). Under 1 V/m-normalized 2-THz radiation incident from the air, the electric field within the 2DEG is amplified approximately 38 times by the dipole antenna on top.

**Figure 5:**
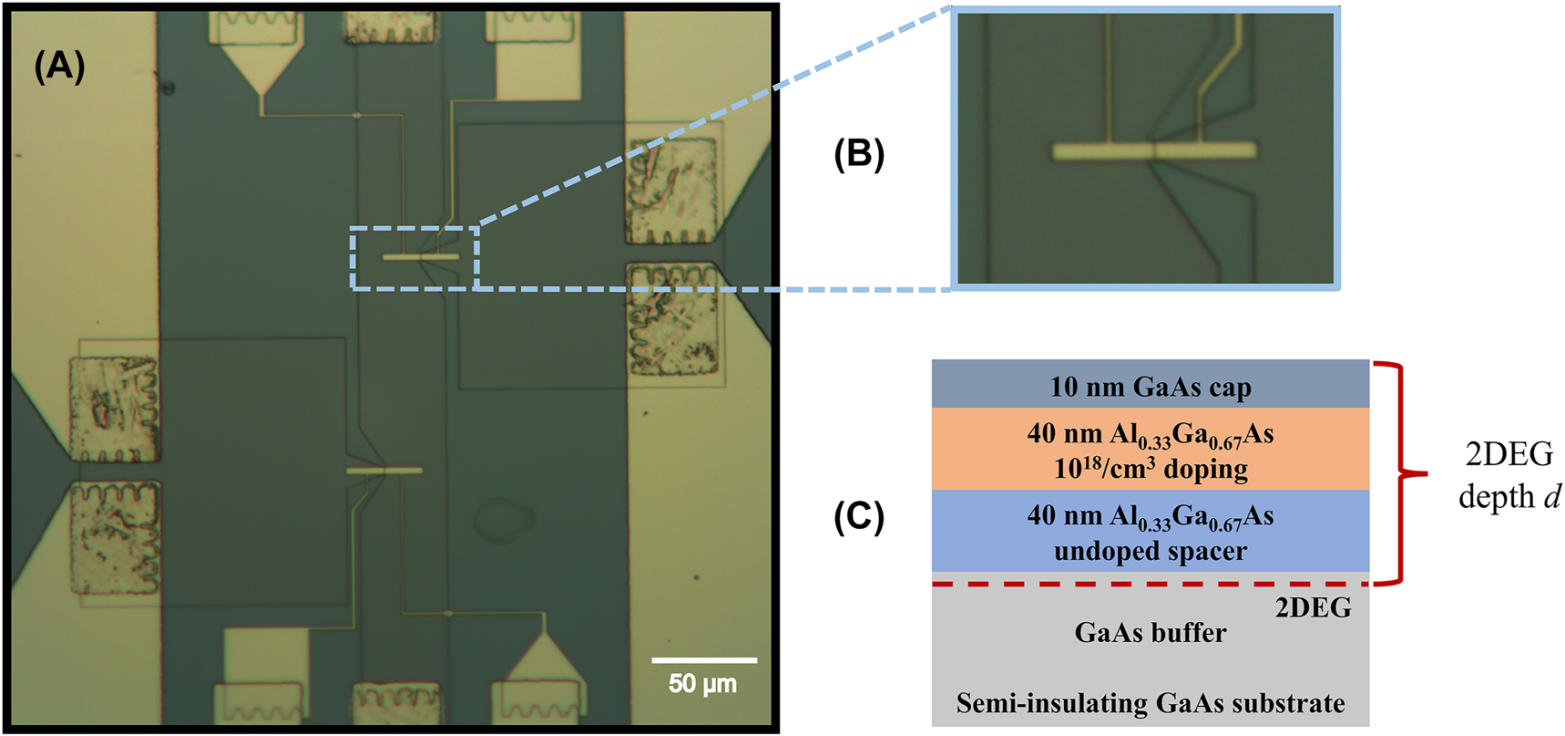
Sample view. (A) Microscope photo of a real sample. (B) Enlarged photo of the active part of a device. (C) Layout of the wafer used for the PETS THz detectors.

The wafer used for device fabrication is grown by molecular beam epitaxy and sequentially consists of a 1-μm undoped GaAs buffer layer, a 40-nm undoped Al_0.33_Ga_0.67_As spacer, a 40-nm n-doped Al_0.33_Ga_0.67_As layer with 10^18^/cm^3^ Si donors and a 10-nm GaAs cap, as shown in Figure 5(C). The 2DEG characterized by Hall measurements features an electron density of 1.52 × 10^11^/cm^2^ and a mobility of 4.3 × 10^5^ cm^2^/Vs in the dark at 1.5 K. The dual-gate field effect transistor at the top comprises a 2DEG mesa, sources, drains and two gates, as shown in Figure 5(A). By wet chemical etching, the 2DEG layer located 90 nm below the surface is shaped into a narrow channel in which electrons are confined. The Ohmic source and drain contacts, made of annealed AuGeNi eutectic alloy, are connected to the 2DEG channel for electron transport. TiAu gates on top of the device are shaped into the form of a dipole antenna by electron beam lithography. The two gates are used to simultaneously apply the dc gate voltages to create a potential step and to focus and amplify the incident THz radiation.

To characterize the PETS THz detector, a 1.9-THz quantum cascade laser (QCL) [42], [43] electrically modulated by a square wave with 772 Hz frequency and 3.86 % duty cycle serves as the source. The QCL wavelength is *λ* = 159.38 µm, as measured by a Fourier-transform infrared spectrometer. The QCL source used in this work, fabricated on a GaAs/AlGaAs substrate using molecular beam epitaxy [44], has the same design as in Ref. [45] but with 4.4 nm instead of 5.0 nm injection barrier thickness. The detector and the QCL are cooled down to 8 K and 16 K, respectively, in two liquid helium continuous flow cryostats. A cylindrical multimode waveguide system [46] with 60 % transmission efficiency connects the two cryostats to deliver the THz radiation from the QCL source to the PETS detector [47]. Using a Thomas Keating absolute power meter, we measure the total incident power at the sample space, and using a Golay cell with a 0.5 mm iris on a set of scanning stages, we capture the intensity distribution across the sample space. This way we determine the maximum time-averaged intensity incident on the sample to be around 2.4 μW/mm^2^.

### 3.2 Photoresponse characterization

**Figure 6:**
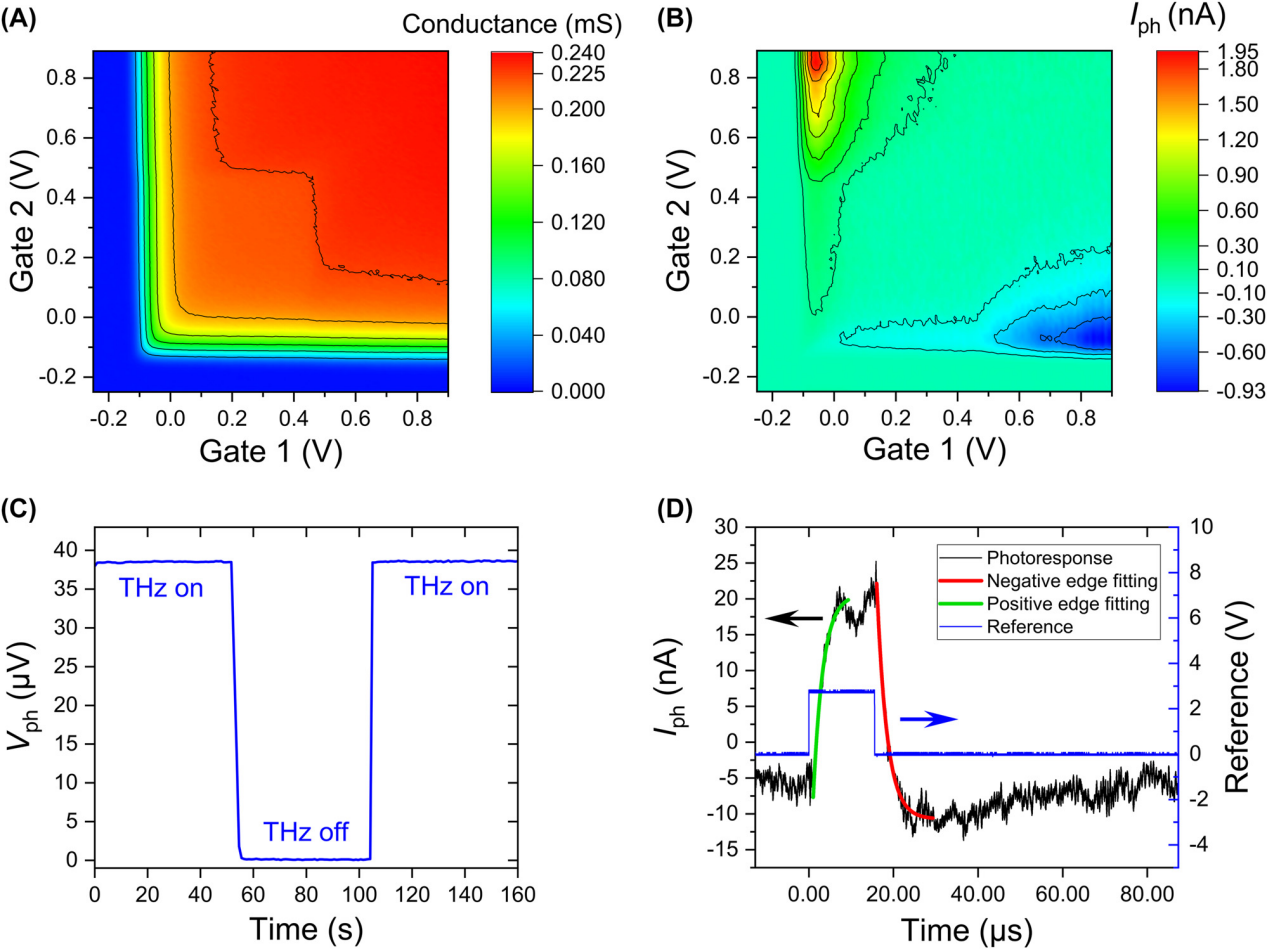
Photoresponse and speed measurement of the dipole antenna PETS THz detector under 1.9-THz radiation with zero source-drain bias. (A) 2D map of conductance. (B) 2D map of source-drain photocurrent response. (C) Source-drain photovoltage measurement with ‘on’, ‘off’ states achieved by mechanically blocking and unblocking the waveguide. The device is cooled down to 8 K during the photoresponse measurement. The source in the setup is a pulsed quantum cascade laser (QCL) with 772 Hz frequency and 3.86 % duty cycle. All data points are collected by lock-in amplifiers. (D) Speed measurement of the device. The black curve is the photocurrent response of the detector to an incident THz pulse which is driven by the blue square wave (1.2 % duty cycle, 772 Hz frequency). The photocurrent response is averaged over 250 sweeps and recorded by a 200 MHz-oscilloscope. The rising edge and the falling edge of the response are fitted to extract the response time of the device.

The conductance of this sample as a function of the two gate voltages is shown in Figure 6(A). The device is driven by a 3-mV sine wave with 86.5 Hz oscillation frequency provided by a lock-in amplifier. As the ratio of current to voltage, the conductance is measured in a two-terminal configuration in which the voltage and the current across the source and the drain in Figure 1(B) are tested by lock-in amplifiers. In the 2D map, the 2DEG channel pinches off and the conductance vanishes at the left and bottom edges. The corresponding threshold voltages are −0.12 V and −0.15 V, respectively.

A strong photoresponse is generated under THz radiation with zero source-drain bias. The 2D map of the photocurrent response as a function of the two gate voltages, *I*_ph_(*G*_1_, *G*_2_), is depicted in Figure 6(B). Considering that the 2DEG channel pinches off under a strong negative gate bias as shown in Figure 6(A) and the signal will be noisy due to the large gate leakage when the gate voltage goes beyond 0.9 V, the range of the two gate voltages is limited to (−0.25 V, 0.9 V). The data are measured with a lock-in amplifier with the QCL modulation frequency, 772 Hz, as a reference, and a current preamplifier with 200 nA/V amplification is employed for the photocurrent measurement.

The photoresponse is approximately antisymmetric with respect to the diagonal line *U*_G1_ = *U*_G2_. The optimal photoresponses are achieved at the top-left and bottom-right corners of the 2D map, where one of the two gates is negatively biased and the other is positively biased. Here, the strong positive gate bias leads to a widening of the conducting channel; this effect is also the reason for the enhanced conductance in Figure 6(A).

The highest source-drain photovoltage response, as shown in Figure 6(C), is 38.5 μV at *U*_G1_ = −0.11 V and *U*_G2_ = 0.85 V with a corresponding resistance of 22.5 kΩ, while the optimal photocurrent achieved here is 1.95 nA at the point (*U*_G1_, *U*_G2_) = (−0.06 V, 0.85 V), which is relatively far away from the pinch-off. The low corresponding impedance of the best photocurrent point, 6.6 kΩ, enables fast photocurrent detection. The on-off states of the photovoltage response in Figure 6(C) are achieved by mechanically blocking and unblocking the copper waveguide. The signal is around zero when the THz radiation is “off” proving that the photoresponse is truly THz-induced.

By normalizing the responsivity to a reference area of *λ* ^2^/4, we can estimate the photovoltage responsivity as 2.5 kV/W. This is higher than that of the broadband bow-tie antenna detector in Ref. [38], which has 1.4 kV/W responsivity, if normalized to the same reference area.

We estimated the theoretically possible magnitude of photoresponse to be 17 nA, see details in Supplementary Information, Section 2, assuming an infinitely sharp potential step without any scattering events in the quantum region.

The thickness of the substrate used in our sample is (500 ± 25) µm, which is substantially thicker than the wavelength of the incident radiation. For a 500 µm GaAs substrate, the resonant frequencies exhibit a Fabry–Perot period of approximately 84.5 GHz. We show in the Supplementary Information, Section 4 and Figure SI-2, the theoretically expected and the experimentally measured transmittance of the same type of wafer as used for the PETS detectors. In our real device, we use a single-side polished wafer with a rough back-side, which will reduce the effect of the Fabry–Perot interference. The maximum-to-minimum ratio of 3.67 in the ideal theoretical Fabry–Perot effect is reduced by ca. 22 % to 2.86 in the experimentally measured data. Therefore, Fabry–Perot interference could affect the electric field distribution, and is one reason for the difference between the measured and the theoretically expected photocurrent value. The limited precision of the known substrate thickness precludes a more accurate estimate of the effect in the real sample.

### 3.3 Response speed characterization

To estimate the response time and the bandwidth of the dipole antenna PETS detector, the THz-induced photoresponse is amplified by a current preamplifier with 800 kHz bandwidth and 2 μA/V amplification and recorded by an oscilloscope with a bandwidth of 200 MHz. The incident THz pulses are from a 1.9-THz QCL modulated by a square wave (the blue line in Figure 6(D)) with 772 Hz frequency and 1.2 % duty cycle. The black line in Figure 6(D) shows the time trace of the THz-induced photocurrent from the dipole antenna PETS detector on a microsecond time scale. To extract the response time, *τ*, we fit the falling edge of the photoresponse time-dependence using an exponential function:

(6) $$I=A+I_{0}\cdot e^{-\left(t-t_{0}\right)/\tau }$$

A similar formula is used to fit the rising edge. The fitted time constants *τ* of the pulse are 2.32 μs (rising time) and 2.41 μs (falling time), corresponding to a bandwidth *BW* = 1/2*πτ* of 69 kHz and 66 kHz, respectively.

The response time of our device is on the order of a few microseconds. As such, our detector is many orders of magnitude faster than thermal detectors, such as Golay cells, thermopiles, pyroelectric detectors, silicon bolometers, and microbolometer arrays, which typically have a response time >1 ms [13], [14], [48], [49]. As a result, PETS detectors enable acquisition of single pulses emitted by the QCL and can be used to study the change in power emitted by the QCL during a terahertz pulse, e.g., due to thermal heating or mode hopping, or embedded in fast feedback circuits for QCL amplitude stabilization [50]. While some FET THz detectors demonstrate significantly faster response times [29], [51] than our current PETS THz detector, our devices hold the potential to operate at much higher speeds. To this end, the device resistance and capacitance need to be reduced. The minimal area of the two gates (not considering connections to the contacts) corresponds to a gate-2DEG capacitance of 0.13 pF, and together with the channel resistance of 4.63 kΩ at the operating point, a theoretical bandwidth of 260 MHz can be expected. In addition, in our design we placed the Ohmic contacts far away from the antenna, with a distance on the order of 100 µm > *λ*/2, in order to exclude any THz electromagnetic field coupling between the Ohmic contacts and the active part of the device containing the THz antenna. However, this large gate-Ohmic distance also introduces an area of 2DEG material with high resistivity. We expect that future device designs with a smaller gate-Ohmic distance will yield substantially higher response speeds, and RF waveguide structures (such as co-planar waveguides) should also be integrated onto the chip in future PETS detectors in order to extract high-bandwidth signals from the active part of the device [52].

### 3.4 Temperature dependence

**Figure 7:**
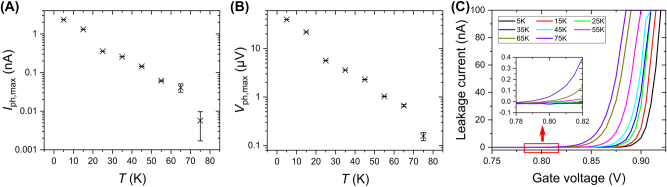
Temperature dependence of PETS THz detectors. The maximum photocurrent (A), photovoltage (B) responses generated by the PETS THz detector at different temperatures, ranging from 5 K to 75 K. The voltage on the narrow gate is 0.8 V, and the wide gate is negatively biased. (C) Current leakage of the positively biased narrow gate at different temperatures. A heater in the cryostat is utilized to stabilize the sample at the target operating temperature, and 40 min are given to allow the signal to achieve a state of stability.

The photoresponse of our dipole antenna detector in Section 3.1 was characterized at 8 K. To investigate the temperature dependence of PETS THz detectors, a device with identical bow-tie antenna design as in Ref. [38] but with a 3-μm instead of 2-μm mesa width was tested at different temperatures. The antenna on top of the GaAs/AlGaAs heterojunction in this device is an asymmetric bow-tie with a right “narrow gate”, which is split in two halves connected by a narrow bridge, and a left “wide gate”, which has no cutout, see Figure SI-3. The narrow gate of the device is positively biased with a voltage of 0.8 V, while the wide gate is biased negatively. At each temperature interval, 40 min were given after setting the temperature setpoint to allow the sample to thermalize and to stabilize the photoresponse before the characterization. For each temperature, the wide gate voltage corresponding to the maximum photocurrent and photovoltage was found, and the photoresponse values maximized as a function of wide gate voltage were recorded and are presented in Figure 7(A) and (B).

As shown in Figure 7(A) and (B), the photoresponse drops down with temperature until 75 K, at which point the signal is of the same order as the noise. The drop-off is approximately exponential with temperature, which suggests a functional form of $I_{\text{ph}}\left(T\right)=I_{\text{ph}}\left(T=0\right)e^{-\xi T}$ . Let us consider the physical reasons of the observed temperature dependence of the photoresponse.

Firstly, the in-plane photoelectric effect itself, which relies on quantum transitions in degenerate electron systems, is not expected to fall off with temperature: when the Fermi level becomes smeared out as the temperature grows, this does not influence the probability of electrons to absorb a photon. Secondly, the degradation of the effect with temperature could be due to the gate leakage. Figure 7(C) illustrates that there is no gate leakage at 0.8 V even at a high operating temperature, 75 K. Therefore, we can exclude a change in gate leakage current as a possible reason for the observed change of the photoresponse with temperature.

The most probable reason for the decay of the photoresponse with temperature in practical PETS THz detectors is the increasing electron scattering rates. As electrons in the gap region absorb photons due to the in-plane photoelectric effect, they acquire elevated energies from *E*_F_ to *E*_F_ + *ℏω*, and then need to move under the gate-covered areas towards the contacts. While the IPPE effect itself is collisionless, this classical motion is accompanied by scattering of electrons with impurities and, at elevated temperatures, with phonons. Physically, an exponential decay of the photoresponse with the length *L* that electrons need to travel can be expected, with the mean free path being the characteristic length scale. This leads to the following phenomenological formula:

(7) $$I_{\text{ph}}\left(T\right)∼Ce^{-L/\lambda_{\text{e}}}=Ce^{-L/\left(v_{\text{F}}\tau \right)}=Ce^{-\frac{Le}{v_{\text{F}}m_{\text{eff}}}\times \frac{1}{\mu }},$$

where *λ*_e_ is the mean free path, *v*_F_ is the Fermi velocity, and *μ* = *eτ*/*m*_eff_ is the mobility. For *L*, we take the barrier length that electrons need to pass under the negatively biased gate in the partially depleted 2DEG. This minimum length that electrons needs to travel to escape the gated region is *L* = 5.6 μm, see Supplementary Information, Section 5 and Figure SI-3. The mobility *μ* is known to decrease with growing temperature. To characterize this effect quantitatively, we use the results obtained in Ref. [53], where it was shown that in GaAs-AlGaAs heterostructures, the temperature dependence of the mobility can be well described by the function 1/*μ* = 1/*μ*_c_ + *αT*, with constants *μ*_c_, *α* > 0. In that case, the linear increase of the inverse mobility with temperature would exactly correspond to an exponential decay of $I_{\text{ph}}\left(T\right)$ :

(8) $$\ln\left(\frac{I_{\text{ph}}}{\text{nA}}\right)=A+MT=\ln\left(\frac{C}{\text{nA}}\right)-\frac{Le}{v_{\text{F}}m_{\text{eff}}}\left(\frac{1}{\mu_{\text{c}}}+\alpha T\right)$$

A fit of this formula to our photocurrent data in Figure 7(A) yields *M* = −0.0685/K (*M* = −0.0687/K for photovoltage). To make a quantitative estimate, we take *v*_F_ = 10^5^ m/s corresponding to a density of 5.4 × 10^10^/cm^2^ expected under the wide gate at the gate voltage of the maximum photoresponse. This yields $\alpha =4.6\times 10^{-8}\;\text{Vs}/\left(\text{c}{\text{m}}^{2}\;\text{K}\right)$ , in good agreement with the values for *α* in the range of $1-5\times 10^{-8}\;\text{Vs}/\left(\text{c}{\text{m}}^{2}\;\text{K}\right)$ in Ref. [53].

The results presented here indicate the operational capability of our PETS THz detectors at relatively high temperatures, up to 75 K. Other processes that may also play a role in the temperature dependence, especially at temperatures above ca. 50 K, are thermionic emission and electron excitation between quantum well and doped regions [54].

## 4 Discussion and conclusion

In summary, we demonstrated a dipole-antenna PETS THz detector based on a GaAs/AlGaAs heterojunction to systematically investigate the dependence of the in-plane photoelectric effect on multiple parameters, including the dimensions of the antenna and the operating temperature. Our numerical simulations provided information on the optimal gap size, 2DEG depth, antenna gate thickness, and the interdependence of these parameters. Based on the simulation results, we fabricated the first PETS detector with a dipole antenna, having a 2DEG depth *d* = 90 nm, gate thickness *h* = 175 nm and gap size *b* = 190 nm. It showed a strong photoresponse (∼1.95 nA) to 1.9-THz radiation under 2.4 μW/mm^2^ incident intensity, confirming successful operation via the in-plane photoelectric effect. Notably, while in Ref. [38] an asymmetric bow-tie antenna was used, with one of the wings having a cut-out that reduced the gate length to 0.2 µm, here we demonstrated a PETS THz detector with a symmetric antenna structure. A qualitatively similar response via the in-plane photoelectric effect is observed, which shows that an asymmetric antenna is not required for the in-plane photoelectric effect to be observed. The response time of our device, ∼2 μs, is significantly faster than thermal detectors, and could be further improved via the reduction of resistance and capacitance, as well as the on-chip integration of RF waveguides.

The photoresponse of our PETS detectors decreases approximately exponentially with the temperature, and shows a capability of operating up to 75 K. The degradation of the photoresponse with the growing temperature is related to the scattering of the photoexcited electrons on their way from the interaction area in the gap between the antenna wings to the contacts. Reducing the distance between the gap and contacts in future sample designs should improve the temperature performance of the detectors. The use of other materials, such as gallium nitride (GaN), can also enhance the temperature performance, due to its much wider bandgap of 3.4 eV [55] compared to GaAs (1.4 eV) [40], which means that devices based on a AlGaN/GaN heterostructure can operate at higher temperatures because the large bandgap reduces the inherent thermal generation of charge carriers.

The PETS detection mechanism relies on the creation of a combined dc and ac potential step. Therefore, one common feature of PETS detectors is the confinement of the incident radiation to a narrow gap region where the conversion into a current/voltage response takes place. A variety of antennas can be employed in combination with PETS detectors. These antennas can be broadband in frequency response (e.g. bow-tie [38]) or resonant, such as the dipole antenna presented here, and can also employ designs with various sensitivity to polarization (e.g. dipole antenna for linear polarization as in this work, or spiral antennas for circular polarization). The overall frequency and polarization response of PETS detectors will be determined by the antenna geometry; however, once the radiation is confined into a narrow gap region, the conversion into a THz photoresponse will still mainly be determined by the functions shown in Figures 3 and 4 (with the exception of a pre-factor proportional to the antenna amplification). Hence, the results of our work provide a universal reference for the design of PETS detectors and pave the way to future high responsivity, fast PETS THz detectors operating at high temperatures.

## Supplementary Material

Supplementary Material Details
