# Supplementary material for: Photoelectric tunable-step terahertz detectors: a study on optimal antenna parameters, speed, and temperature performance
Source: Nanophotonics. 2024 Feb 1;13(10):1917–28. doi: 10.1515/nanoph-2023-0864 (PMC11501986; doi:10.1515/nanoph-2023-0864)
Supplement: Supplementary file 1 — Supplementary Material Details [file j_nanoph-2023-0864_suppl_001.pdf]

Supplementary Information to:

Ran Chen*, Ruqiao Xia, Jonathan Griffiths, Harvey E. Beere, David A. Ritchie, Wladislaw Michailow†

Photoelectric tunable-step terahertz detectors: a study on optimal antenna parameters, speed, and temperature performance

1 Contribution of the vertical electric field components

We also calculated the vertical, z-component of the electric field, to compare with Fig. 3(A). In Fig. SI-1 (A), we show the distribution of the z-component of the electric field along the x-axis, at a depth of d = 90 nm under the surface, for various values of b. In Fig. SI-1 (B), we calculated the radiation-induced ac gate-to-channel voltage:

$$U_{\mathrm{ac,z}}(b,d) = \int_{-d}^{0} E_{\mathrm{z}}\left(\mathrm{x} = -\frac{b}{2}, z\right) \mathrm{dz}$$

By comparing the plots to Fig. 3 (A) and (C), one can see that the contribution of the vertical, z-component of the electric field is much smaller than the contribution of the horizontal, x-component: both $U_{\mathrm{ac,z}}$ and $|E_{\mathrm{z}}|$ are notably smaller than $U_{\mathrm{ac,x}}$ and $|E_{\mathrm{x}}|$ for the parameter region of b and d that is relevant for PETS detectors as described in the main text.

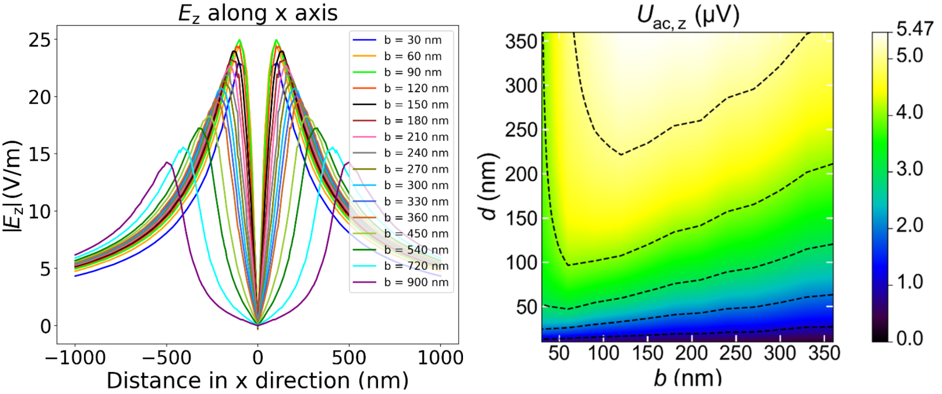

Figure SI-1: Evaluation of the vertical, z-component of the electric field. (A) Absolute value of the electric field in z-direction at a depth $d = 90\ \mathrm{nm}$ under the surface. (B) Radiation-induced ac-potential difference, $U_{\mathrm{ac,z}}(b,d)$, calculated by integrating E_{z} along the z direction from 0 to -d as a function of the gap size b and the 2DEG depth d.

2 Theoretical estimation of the photoresponse due to the in-plane photoelectric effect

From Eq. (4), we can estimate the theoretically expected photoresponse in the in-plane photoelectric effect according to Eq. (4) in the main paper and Refs. [38, 39]. For the fabricated device parameters, the 1 V/m-normalised $U_{\mathrm{ac,x}}$ is 15.2 μV, see Fig. 4(F). The incident intensity of 2.4 μW/mm² at the sample space yields 30 V/m incident electric field, which correspondingly amplifies $U_{\mathrm{ac,x}}$ giving a value of 0.459 mV. The lithographically defined channel width of 4 μm is expected to be reduced to ca. 2.7 μm taking into account edge depletion effects, which are expected to reduce the channel width by ca. 0.65 μm on either side based on previous measurements of these types of samples. From the conductance data shown

*Corresponding author: Ran Chen, Cavendish Laboratory, University of Cambridge, CB3 0HE Cambridge, UK, E-mail: rc830@cam.ac.uk
†Corresponding author: Wladislaw Michailow, Cavendish Laboratory, University of Cambridge, CB3 0HE Cambridge, UK, E-mail: wm297@cam.ac.uk
Ruqiao Xia, Jonathan Griffiths, Harvey E. Beere: Cavendish Laboratory, University of Cambridge, CB3 0HE Cambridge, UK. David A. Ritchie: Cavendish Laboratory, University of Cambridge, CB3 0HE Cambridge, UK; Swansea University, Singleton Park, Sketty, Swansea SA2 8PP, UK.

in Fig. 6(A), we estimate the threshold voltage U_{th} by fitting the measured conductance as a function of gate 1-voltage with zero gate 2-voltage, and find $U_{th} \approx -0.11$ V. Using the GaAs dielectric permittivity of 12.6 and an approximate 2DEG-surface distance of 90 nm, we find the equilibrium density to be $8.6\times10^{10}/\text{cm}^2$, corresponding to an equilibrium chemical potential of 3.1 meV. At the point of maximum photoresponse, (U_{G1}, U_{G2}) = (-0.06V, 0.85V), the left and right chemical potentials normalized to the photon energy $\hbar\omega \approx 7.8$ meV are $\mu_1/\hbar\omega = 0.18$ and $\mu_2/\hbar\omega = 3.4$, respectively. At these values, the function J in Eq. (4) in the main paper equals 0.158 [57], yielding 46 nA of internal photocurrent of the quantum-mechanical current source representing the THz-illuminated potential setup. The internal resistance of the quantum-mechanical current source is 2.47 kΩ. Taking into account the resistive loading of the quantum-mechanical current source by the measured classical resistances, we get a theoretically expected photocurrent of 17 nA.

3 Theoretical estimation of the photoresponse due to the plasmonic mixing

We can compare the theoretically expected photoresponse due to plasmonic mixing as $U_{ph} = eU_{ac,z}^2/(4\ \eta\ k_B T)$ from Ref. [16]. From the conductance data in Fig. 6(A), we estimate the ideality factor, $\eta \approx 13 - 16$. To obtain the maximum possible value for the photovoltage induced by the plasmonic mixing, we take the smallest ideality factor of 13, and obtain the value for the ac-induced gate-to-channel voltage $U_{ac,z}$ in section 1. At the corresponding temperature, this yields an expected photovoltage due to plasmonic mixing of $U_{ph} \approx 0.25$ µV. Due to the back-to-back connection of two 2DEGS with different densities, the residual plasmonic mixing voltage will be the difference between the contributions of the left and right parts of the 2DEG, so in practice, the plasmonic contribution to the response will be even less. Even if instead of $U_{ac,z}$ we take the same value we used for $U_{ac,x}$ in section 2 above, we get $U_{ph} \approx 5.2$ µV, which is still much smaller than the experimentally measured 38.5 µV.

4 Measurement of the Fabry-Perot effect

We measured the Fabry-Perot effect on a single-side polished GaAs wafer of the same type as was used for the fabrication of the PETS THz detectors using THz time-domain spectroscopy system from Menlo Systems, Tera K15. The result is shown in Fig. SI-2 (B). For comparison, Fig. SI-2 (A) shows the theoretically calculated Fabry-Perot effect in the transmittance, assuming two ideally flat surfaces on a 500 µm thick substrate with refractive index 3.55. The ratio of maximum to minimum is 3.67 in the theoretical case, and 2.86 in the experimental data around 1.9 – 2.0 THz.

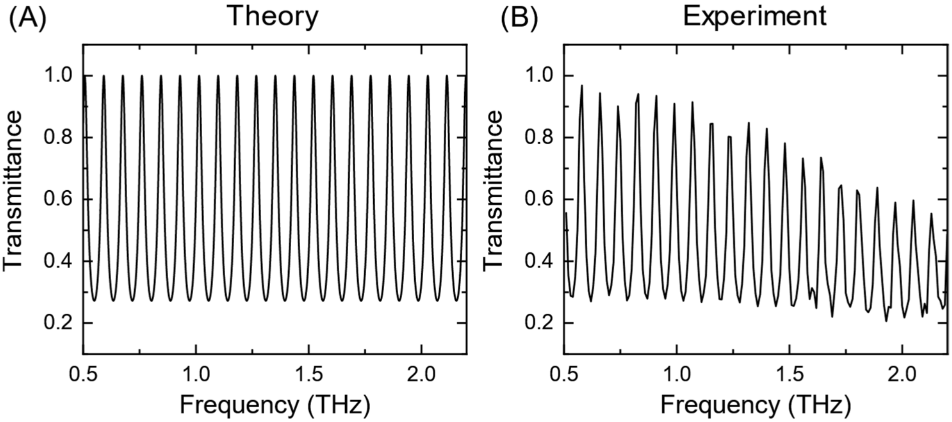

Figure SI-2: Evaluation of the Fabry-Perot effect. (A) Theoretical calculation of the transmittance for a 500 µm thick GaAs substrate with refractive index 3.55, (B) Measured data using terahertz time-domain spectroscopy.

5 Geometry of the bow-tie antenna detector

Figure SI-3 shows the geometry of the bow-tie antenna detector used to measure the temperature dependence. The lithographically defined channel width is 3 µm. The length L = 5.6 µm used in the analysis is indicated. It corresponds to the smallest distance that electrons need to pass from the center of the gap region to escape the region under the wide gate. The black contour shows the lithographically defined mesa edge, while the blue area indicates the area where the 2D electron gas is expected to be located. It is offset inwards by 0.65 µm, a value representing the approximate sidewall etching and edge depletion based on previous measurements on these types of samples.

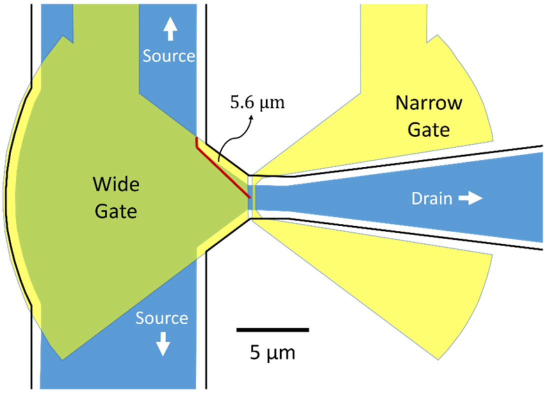

Figure SI-3: Geometry of the bow-tie antenna detector. Semitransparent yellow: gate metal, black: lithographically defined mesa edge, blue: area where the 2D electron gas is present.
